# Urine dipsticks are not accurate for detecting mild ketosis during a severely energy restricted diet

**DOI:** 10.1002/osp4.432

**Published:** 2020-06-10

**Authors:** Alice. A. Gibson, Elif. I. Eroglu, Kieron. Rooney, Claudia. Harper, Sally. McClintock, Janet. Franklin, Tania. P. Markovic, Radhika. V. Seimon, Amanda. Sainsbury

**Affiliations:** ^1^ Boden Collaboration for Obesity, Nutrition, Exercise and Eating Disorders, Faculty of Medicine and Health The University of Sydney Camperdown New South Wales Australia; ^2^ The Menzies Centre for Health Policy, School of Public Health, Faculty of Medicine and Health The University of Sydney Camperdown New South Wales Australia; ^3^ Discipline of Exercise and Sport Science, Faculty of Medicine and Health, Charles Perkins Centre University of Sydney Sydney New South Wales Australia; ^4^ Metabolism & Obesity Services Royal Prince Alfred Hospital Camperdown New South Wales Australia; ^5^ School of Human Sciences, Faculty of Science The University of Western Australia Crawley Western Australia Australia

**Keywords:** ketogenic diet, meal replacement, very low calorie diet, very low energy diet

## Abstract

**Background:**

Detection of the mild ketosis induced by severely energy‐restricted diets may be a clinically useful way to monitor and promote dietary adherence. Mild ketosis is often assessed using urine dipsticks, but accuracy for this purpose has not been tested.

**Objective:**

To determine the accuracy of urine dipsticks to detect mild ketosis during adherence to a severely energy‐restricted diet.

**Methods:**

Two hundred and sixty three (263) fasting urine and 263 fasting blood samples were taken from 50 women (mean [standard deviation, SD] age 58.0 [4.3] years and body mass index 34.3 [2.4] kg/m^2^) before and at six time points during or for up to 10 weeks after 16 weeks of severe energy restriction, achieved with a total meal replacement diet. The amount of ketones (acetoacetate) in the urine was classified as ‘0 (Negative)’, ‘+/− (Trace)’, ‘+ (Weak)’ or ‘++ (Medium)’ by urine dipsticks (Ketostix, Bayer). The concentration of ketones (β‐hydroxybutyrate) in the blood was measured with our reference method, a portable ketone monitor (FreeStyle Optium, Abbott). The diagnostic accuracy of the urine dipsticks was assessed from the percent of instances when a person was actually ‘in ketosis’ (as defined by a blood β‐hydroxybutyrate concentration at or above three different thresholds) that were also identified by the urine dipsticks as being from a person in ketosis (the percent ‘true positives’ or sensitivity), as well as the percent of instances when a person was not in ketosis (as defined by the blood monitor result) was correctly identified as such with the urine dipstick (the percent ‘true negatives’ or specificity). Thresholds of ≥0.3mM, ≥0.5mM or ≥1.0mM were selected, because mean blood concentrations of β‐hydroxybutyrate during ketogenic diets are approximately 0.5mM. Sensitivity and specificity were then used to generate receiver operating characteristic curves, with the area under these curves indicating the ability of the dipsticks to correctly identify people in ketosis (1 = perfect results, 0.5 = random results).

**Results:**

At threshold blood β‐hydroxybutyrate concentrations of ≥0.3mM, ≥0.5mM and ≥1.0mM, the sensitivity of the urine dipsticks was 35%, 52% and 76%; the specificity was 100%, 97% and 78%; and the area under the receiver operating characteristic curves was 0.67, 0.74 and 0.77, respectively. These low levels of sensitivity mean that 65%, 48% or 24% of the instances when a person was in ketosis were not detected by the urine dipsticks.

**Conclusion:**

Urine dipsticks are not an accurate or clinically useful means of detecting mild ketosis in people undergoing a severely energy‐restricted diet and should thus not be recommended in clinical treatment protocols. If monitoring of mild ketosis is indicated (eg, to monitor or help promote adherence to a severely energy‐restricted diet), then blood monitors should be used instead.

## 1 INTRODUCTION

Severely energy‐restricted diets have been in clinical use for over 40 years and are the most intensive and effective dietary treatment for obesity.^1^, ^2^ Severely energy‐restricted diets are a broad term used to describe very low energy diets (VLEDs, <3,300 kJ or 800 kcal per day) and low energy diets (LEDs, <5,000 kJ or 1,200 kcal per day). VLEDs and LEDs often involve total or partial replacement of food intake with specially formulated meal replacement products.^3^, ^4^ Despite such a severe restriction of energy intake, adherence is surprisingly high with severely energy‐restricted diets, as evidenced by the rapid and substantial weight loss achieved.^5^, ^6^, ^7^ High adherence to the severe restriction of energy intake is thought to be due at least in part to the appetite supressing effect shown to be associated with these diets.^8^, ^9^ This appetite‐suppressing effect may be due to the associated mild ketosis.^10^, ^11^ Ketosis is a condition where hepatic production of ketone bodies or ‘ketones’ (β‐hydroxybutyrate, acetoacetate and acetone) is increased, leading to elevations in their concentrations in various body fluids. Ketosis is a coordinated metabolic response that provides ketones as an alternative fuel source for the brain and other organs when glucose is in short supply. The mild ketosis associated with a severely energy‐restricted diet, where blood β‐hydroxybutyrate concentrations rise to an average of about 0.5mM,^10^ is distinct from the life‐threatening state of ketoacidosis (where blood β‐hydroxybutyrate concentrations are >3mM), which occurs only in people with diabetes and is accompanied by concomitant hyperglycaemia and a decrease in plasma pH.^12^ Given the purported association between mild ketosis and appetite suppression, the detection of ketosis during a severely energy‐restricted diet may be a clinically useful way to monitor and promote adherence.

A strong body of evidence suggests that higher level of adherence to an energy‐restricted diet, regardless of the type of diet, is an important factor in weight loss success.^13^, ^14^, ^15^, ^16^ It follows then that strategies that help to promote adherence to an energy‐restricted dietary intervention could improve weight loss success. There are numerous factors that may influence an individual's level of adherence to an energy‐restricted diet, and clinicians and consumers will likely need access to a range of strategies to promote adherence. In energy‐restricted dietary interventions, adherence is commonly monitored by recording food intake or body weight. As well as monitoring adherence, these methods are thought to help promote adherence, and thereby weight loss. To our knowledge, no studies have investigated whether monitoring adherence by assessing ketosis during a severely energy‐restricted diet, or to other ketogenic diets, promotes adherence.

There are two types of testing commercially available for monitoring ketosis—urine dipsticks and blood monitors. Urine dipsticks detect the presence of acetoacetate in urine by a colorimetric reaction with nitroprusside. Urine dipsticks have been in clinical use for more than 50 years, initially to detect life‐threating ketoacidosis in individuals with diabetes.^17^ In contrast, blood monitors detect the concentration of β‐hydroxybutyrate in blood, typically capillary blood from a finger prick. Blood monitors have overcome some of the limitations of urine dipsticks, most notably that urine dipsticks only provide a surrogate marker of the more clinically relevant β‐hydroxybutyrate, as well as the difficulty of obtaining a urine sample from an unconscious patient. Blood ketone monitors have been commercially available to the general public for approximately 10 years,^17^ but they remain predominantly used by clinicians rather than consumers.

There are a number of reasons why clinicians or consumers with diabetes may prefer to use urine dipsticks instead of blood monitors. Compared to blood monitors, urine dipsticks are equally able to identify ‘true positive’ cases of ketoacidosis in people with diabetes (high sensitivity), albeit they have a lower ability to identify ‘true negatives’ among people with diabetic ketoacidosis (lower specificity).^17^, ^18^, ^19^, ^20^ As well as being less invasive than blood ketone monitors (some people may be averse to pricking their finger), urine dipsticks are at least seven times cheaper than blood monitors, at around US$0.10 per dipstick compared with around US$0.70 per test trip for the blood monitors. Furthermore, the blood ketone monitor itself typically costs at least another US$25.00.

In light of the above‐mentioned benefits of urine dipsticks over blood monitors for gauging ketosis, some clinical treatment protocols and resources for severely energy‐restricted diets administered using total^21^ or even partial^22^ meal replacement recommend the use of urine dipsticks as a means of monitoring or promoting adherence to the diet. However, no studies have examined the accuracy of urine dipsticks in detecting the much lower levels of ketosis that are associated with severely energy‐restricted diets in a nondiabetic population. If low ketone levels are undetectable in urine, then use of urine dipsticks may result in false negative results. That is, a urine dipstick may not indicate the presence of ketones in the urine despite a person being in mild ketosis according to the presence of ketones in their blood. This has important implications for persons following severely energy‐restricted diets, as well as for clinicians monitoring adherence in their patients. Therefore, the aim of this study is to compare the ability of a urine dipstick to detect ketones in urine in people with varying levels of ketones present in the blood during a severely energy‐restricted diet.

## 2 METHODS

### 2.1 Participants

This is a substudy of the TEMPO Diet Trial (Type of Energy Manipulation for Promoting optimum metabolic health and body composition in Obesity), a randomized controlled trial comparing the long‐term effects of severe energy restriction versus moderate energy restriction in postmenopausal women, aged 45 to 65 years, with obesity (body mass index 30‐40 kg/m^2^).^23^, ^24^, ^25^ Participants were at least 5 years postmenopause at the time of recruitment and were required to be sedentary (ie, doing less than 3 h of structured physical activity per week). Participants with osteoporosis or diabetes, and those taking medication affecting body composition, were excluded. The full inclusion and exclusion criteria and the rationale for these have been detailed in our published protocol.^25^ The trial was approved by the Sydney Local Health District Ethics Committee (Royal Prince Alfred Hospital Zone) and registered with the Australia and New Zealand Clinical Trials Registry (number 12612000651886). All participants provided informed, written consent prior to participation.

This substudy only included the 50 participants who had been randomized to the severely energy‐restricted diet arm of the trial. These women had a mean (standard deviation [SD]) age of 58.0 (4.3) years and body mass index of 34.3 (2.4) kg/m^2^ at baseline (ie, week 0, prior to commencement of the severely energy‐restricted diet).

### 2.2 Severely energy‐restricted diet

Details of the study protocol and 12‐month intervention for the severely energy‐restricted arm of the TEMPO Diet Trial have been reported in detail previously.^23^, ^24^, ^25^ Briefly, participants were randomized to 4 months (16 weeks) of severe energy restriction of 65% to 75% relative to estimated energy expenditure, using a total meal replacement diet, followed by moderate energy restriction of 35% to 25% for an additional 8 months (36 weeks). The total meal replacement products used in the severely energy‐restricted diet provided between 2,630 (630 kcal) and 3,260 kJ (780 kcal) per day in this group of participants. Participants were also allowed to consume two cups of non‐starchy vegetables and a teaspoon of oil each day.

### 2.3 Assessment of ketones in blood and urine

This analysis includes data from baseline (week 0) and from weeks 1, 4, 16, 17 and 26 after commencement of the 16‐week severely energy‐restricted diet. This choice of time points was to provide a range of levels of ketosis, including at time points when participants should theoretically not be in ketosis according to the study protocol (ie, at weeks 0 and 26). At each of these time points, fasting urine and blood samples were collected from participants at the research facility. Participants were asked to urinate before leaving home and then to refrain from urinating until a sample was taken upon arrival at the research facility. A urine dipstick (Ketostix, Bayer) was used to determine the presence or absence of ketones (acetoacetate) in the urine by comparing the colour of the dipstick, after the participants' urine had been pipetted onto the dipstick, against the colours on the back of the packet. The results were classified as either 0 (Negative), +/− (Trace), + (Weak) or ++ (Medium). Fasting concentrations of β‐hydroxybutyrate in blood were determined by allowing a drop of venous blood from a cannula to fall onto the test strip of a blood ketone monitor (FreeStyle Optium, Abbott Australasia Pty Ltd, Victoria, Australia). There were 263 data points in which participants had both a urine dipstick and blood monitor ketone measurement that could be used for analysis in the present study.

### 2.4 Statistical analysis

The accuracy of urine dipsticks to detect ketosis was determined by calculating sensitivity and specificity, which were used to generate receiver operating characteristic curves. Sensitivity is the ability of the urine dipstick (test method) to detect the presence of acetoacetate in the urine when a person is in ketosis, as defined by their blood β‐hydroxybutyrate concentration (measured using the blood ketone monitor, the reference method) being above a particular threshold. In other words, sensitivity is the proportion of ‘true positives’ that are detected by the urine dipstick. Specificity is the ability of the urine dipstick to not detect the presence of acetoacetate in the urine when a person is not in ketosis, as defined above. That is, specificity is the proportion of ‘true negatives’ that are detected by the urine dipstick. A receiver operating characteristic curve is a graphical representation of the relationship, or trade‐off, between sensitivity (shown on the y‐axis) and specificity (represented on the x‐axis as 100‐specificity). The greater the area under the receiver operator curve, the greater the likelihood that the urine dipstick will detect 100% of true positives and 100% of true negatives, with 0% false positives and 0% false negatives (a perfect diagnostic test). An area under the receiver operating characteristic curve of 0.5 means that there is a not only 50% chance that the urine dipstick will detect true positives and true negatives but also a 50% chance it will detect false positives and false negatives (random results). Thus, the greater the area under the receiver operating characteristic curves (or the closer it is to 1), the more accurate the diagnostic test. In this study, the area under the receiver operating characteristic curve tells us how accurate the urine dipstick is at identifying whether or not someone is in ketosis.

The blood ketone monitor was considered to be the reference method as it has been shown to be highly accurate in comparison with laboratory‐determined blood β‐hydroxybutyrate concentrations.^18^, ^19^ Three different thresholds of blood β‐hydroxybutyrate concentrations were used to define whether or not a person was in ketosis: ≥0.3mM, ≥0.5mM and ≥1.0mM. The rationale for this is that although the average blood concentrations of β‐hydroxybutyrate for individuals during a severely energy‐restricted diet has been shown to be approximately 0.5mM,^10^ other research has shown appetite suppression in association with lower levels of ketosis (~0.3mM) during a severely energy‐restricted diet.^8^ Therefore, it would be clinically relevant to be able to determine the ability of the urine dipsticks to act as a diagnostic for ketosis using both of these concentrations as thresholds for ketosis. A higher threshold of ≥1.0mM blood β‐hydroxybutyrate was also included, because these concentrations are not uncommon during a severely energy‐restricted diet in healthy individuals,^10^ and because it enabled determination of whether the accuracy of the urine dipsticks to detect ketosis would be influenced by this higher threshold. The sensitivity and specificity of the urine dipsticks were calculated for each of the three different thresholds (≥0.3mM, ≥0.5mM and ≥1mM). For statistical analysis, the absence and presence of ketosis were defined as ‘0’ and ‘1’, respectively. Statistical analysis was performed with software from MedCalc Software Ltd (Ostend, Belgium).

## 3 RESULTS

**FIGURE 1:**
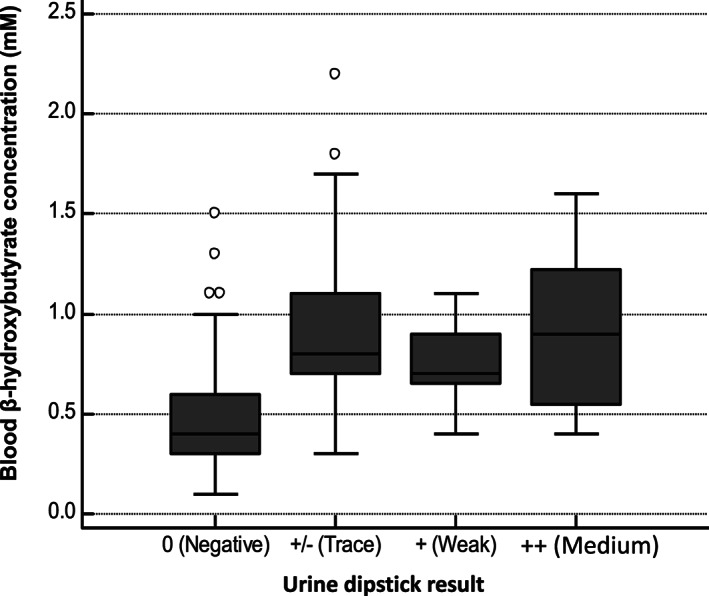
The distribution of blood β‐hydroxybutyrate concentrations measured using a blood ketone monitor (FreeStyle Optium, Abbott), according to whether acetoacetate was detected in the urine using a urine dipstick (Ketostix, Bayer). Boxes represent the 25th and 75th percentiles of the distribution around the median. Whiskers represent 1.5 times the interquartile range, and isolated points represent values lying outside this range

Of the 263 blood samples, 44 (16.7%) had a β‐hydroxybutyrate concentration of <0.3mM, 81 (30.8%) had ≥0.3 to <0.5mM, 104 **(**39.5%) had ≥0.5mM and 34 (13%) had ≥1.0mM β‐hydroxybutyrate concentrations. Figure 1 shows the average blood β‐hydroxybutyrate concentrations according to whether the urine dipstick detected ketones in the urine or not, and at what approximate concentration. In urine samples with no detectable acetoacetate (*n* = 187, 71.1%), the mean blood β‐hydroxybutyrate concentrations were 0.45 (95% CI: 0.40 to 0.47) mM. In the 76 urine samples that had acetoacetate detected at levels of +/− (Trace), + (Weak) and ++ (Medium) (*n* = 60, 77.6%; *n* = 12, 15.8% and *n* = 5, 6.6%, respectively) the mean blood β‐hydroxybutyrate concentrations were 0.92 (95% confidence interval [CI], 0.83‐1.02) mM, 0.75 (95% CI, 0.62‐0.88) mM and 0.92 (95% CI: 0.34 to 1.50) mM, respectively. This demonstrates that although there was an up to two‐fold difference in the blood β‐hydroxybutyrate concentration at times when urine did and did not have acetoacetate detected, there was no clear relationship between approximate concentration of acetoacetate in the urine and blood β‐hydroxybutyrate concentrations.

Table 1 shows the sensitivity and specificity of the urine dipsticks to detect ketosis at the three different thresholds of blood β‐hydroxybutyrate concentrations used to define a state of ketosis. These data show that at blood β‐hydroxybutyrate levels of ≥0.3mM, ≥0.5mM and ≥1.0mM, the sensitivity of the urine dipstick was approximately 35%, 52% and 76%, respectively. In other words, the urine dipsticks correctly identified only 35% to 76% of the individuals who were in ketosis, as defined by their blood β‐hydroxybutyrate concentrations being ≥0.3 to ≥1.0mM. In contrast to this low sensitivity (low rate of true positives), the urine dipsticks had high specificity. With ketosis defined as blood β‐hydroxybutyrate levels of ≥0.3mM, ≥ 0.5mM and ≥1.0mM (or in other words, lack of ketosis being defined as <0.3mM, <0.5mM or <1.0mM), the specificities of the urine dipsticks were approximately 100%, 97% and 78%, respectively (Table 1). In other words, the urine dipsticks correctly identified 78% to 100% of the individuals who were not in ketosis, as determined by their blood β‐hydroxybutyrate concentrations being <1.0 to <0.3mM.

**TABLE 1:** Sensitivity and specificity of the urine dipstick (Ketostix, Bayer) to detect the presence of acetoacetate in the urine at three different thresholds of ketosis, as defined by the blood β‐hydroxybutyrate concentrations shown in the left‐most column

Ketosis threshold	Sensitivity	Specificity
≥0.3mM	34.7 (95% CI, 28.4‐41.4)%	100 (95% CI, 92.0‐100.0)%
≥0.5mM	52.2 (95% CI, 43.5‐60.7)%	96.8 (95% CI, 92.0‐99.1)%
≥1.0mM	76.5 (95% CI, 58.8‐89.3)%	78.2 (95% CI, 72.2‐83.3)%

**FIGURE 2:**
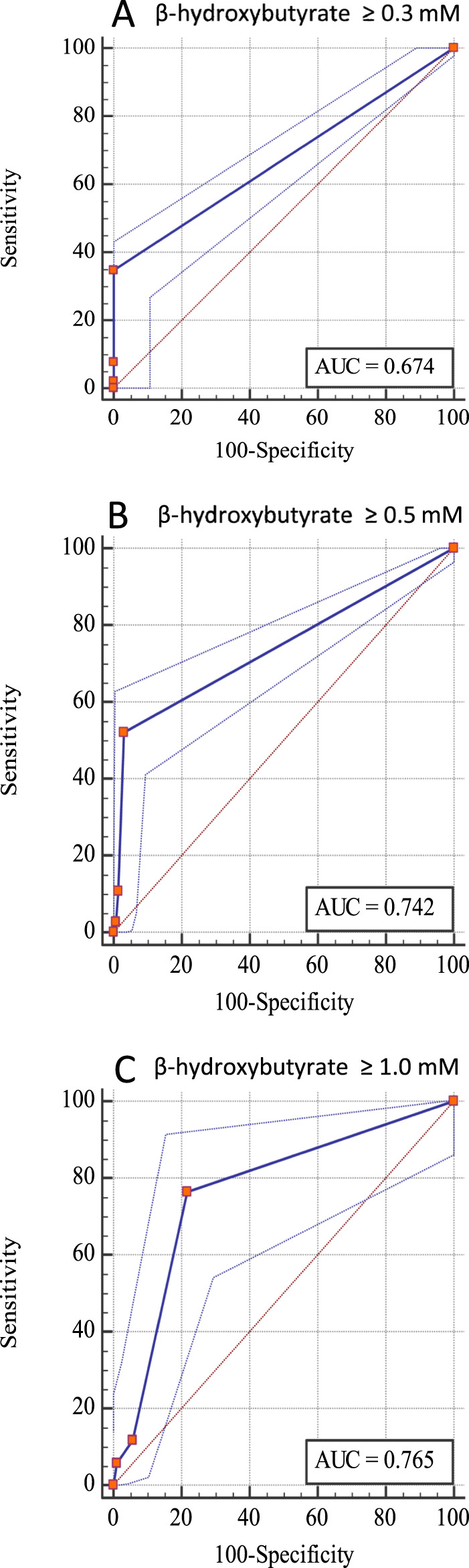
Receiver operating characteristic curves for the detection of ketosis (at three different thresholds of blood β‐hydroxybutyrate concentrations) from acetoacetate detected with a urine dipstick (Ketostix, Bayer). (A) β‐hydroxybutyrate ≥0.3mM, (B) β‐hydroxybutyrate ≥0.5mM and (C) β‐hydroxybutyrate ≥1.0mM. *N* = 263. AUC, area under the curve

Figure 2 shows the sensitivity and specificity for each of the three different thresholds of blood β‐hydroxybutyrate concentrations used to define ketosis, as receiver operating characteristic curves. The area under the receiver operating characteristic curves increased slightly with higher thresholds for ketosis (ie, from 0.67 for blood β‐hydroxybutyrate ≥0.3mM, to 0.77 for blood β‐hydroxybutyrate ≥1.0mM). An area under the curve of 0.67 corresponds to a 67% chance of detecting true positives and true negatives, whereas a value of 0.77 corresponds to a 77% chance of detecting true positives and true negatives. This means that the urine dipsticks were slightly more accurate at detecting ketosis as defined as ≥1.0mM blood β‐hydroxybutyrate concentrations than at ≥0.5 or ≥0.3mM.

## 4 DISCUSSION

This study showed that urine dipsticks are not an accurate or clinically useful way of detecting mild ketosis in people undergoing severely energy‐restricted diets. Urine dipsticks had low sensitivity for identifying when participants were in mild ketosis (as defined by blood β‐hydroxybutyrate concentrations being ≥0.3 and ≥0.5mM), because in only 35% and 52% of those occasions, respectively, did their urine test positive for the presence of ketones with the dipstick. This means that for 48% to 65% of the times when participants were actually in ketosis, as defined by their blood β‐hydroxybutyrate concentrations being at or over these 0.3 and 0.5mM thresholds, the urine dipstick indicated that they were not in ketosis. That is, the rate of ‘false negative’ results were 48% to 65%. Even at a higher threshold for defining ketosis (ie, ≥1.0mM β‐hydroxybutyrate in blood), urine dipsticks still had unacceptably low sensitivity: They only detected the presence of acetoacetate in 76% of cases. This means that 24% of the times when participants had a blood β‐hydroxybutyrate concentration of ≥1.0mM, the highest level normally associated with severely energy‐restricted diets,^10^ the urine dipstick result suggested that they were not in ketosis at all (false negative). A false negative rate of 24% to 65% is not good enough for a test used in clinical practice. These data indicate that urine dipsticks should not be recommended in clinical treatment protocols to monitor or promote adherence to a severely energy‐restricted diet.

Our findings extend those of previous studies, which have compared the accuracy of urine dipsticks with blood ketone monitors to diagnose ketoacidosis in people with diabetes.^20^ A recent systematic review of this topic identified five studies—three of which showed urine dipsticks to have high sensitivity (≥89%, range 89% to 98%) and variable specificity (35% to 100%).^20^ In one of the other two of five studies, sensitivity of the urine dipsticks was low (33%), but specificity was high (94%), and in the remaining study, sensitivity and specificity were not reported.^20^ In contrast, in the present study of participants with varying states of mild ketosis, the sensitivity of the urine dipsticks was low, but the specificity was high. Therefore, although urine dipsticks may have been found to be acceptably accurate in the context of detecting diabetic ketoacidosis in the majority of studies, the current study has shown that they are not accurate at low levels of ketosis.

Our finding that urine dipsticks are not accurate at detecting mild levels of ketosis in people without diabetes is likely explained by the fact that urine dipsticks detect different ketones (acetoacetate) from those detected by blood monitors (β‐hydroxybutyrate), and these ketones differ in their rates of production, utilization and excretion (see Laffel^12^ for an in‐depth review of ketone physiology). For instance, the ketone ratio (defined as the ratio of blood β‐hydroxybutyrate to blood acetoacetate concentrations) increases during ketosis, as β‐hydroxybutyrate is produced in greater quantities than acetoacetate.^12^ It appears therefore that while during diabetic ketoacidosis, the production of acetoacetate appears to be sufficient to enable detection of ketones in the urine, during mild ketosis, acetoacetate is not produced in sufficient quantitates to be consistently detectable in the urine.

If urine dipsticks should not be used to monitor or promote adherence to a severely energy‐restricted diet, does this mean that blood ketone monitors, which provide an accurate indication of mild ketosis,^18^ should be used? Putting aside issues of invasiveness (some people may be averse to pricking their finger for capillary blood collection) and cost (some people may not wish to spend money on them), the outstanding question is whether monitoring adherence to severe energy restriction promotes adherence to severe energy restriction. The literature surrounding the benefits of other forms of self‐monitoring—such as recording food intake or measuring body weight, which have proven benefits for promoting adherence—suggests that it might be useful to monitor blood β‐hydroxybutyrate concentrations using blood monitors. In our clinical experience, some people find it motivating to monitor an outcome other than body weight, particularly one that is a sign of ‘fat‐burning’. Whether measuring blood β‐hydroxybutyrate concentrations using blood monitors during a severely energy‐restricted diet can help promote adherence to the diet, and thereby increase weight loss success, warrants further investigation. In the meantime, the use of blood monitors to monitor ketosis during a severely‐energy restricted diet could be discussed with individuals as an option for monitoring and promoting adherence to potentially improve weight loss success.

There are a number of limitations to this study that should be acknowledged. Firstly, samples came from a relatively small number of people (*n* = 50), who were all postmenopausal women with obesity. Although this could also be seen as a strength due to lack of studies conducted in populations without diabetes, leaner, younger or male individuals could have different metabolic fuel utilization resulting in different ratios of β‐hydroxybutyrate to acetoacetate, and this could potentially affect the accuracy of these tests. Secondly, a portable blood monitor was used to measure blood ketone levels rather than laboratory‐based enzymatic methods, which may have been the ultimate reference method. However, previous research has shown blood β‐hydroxybutyrate monitors to be highly accurate compared with laboratory enzymatic analysis. For instance, in one study among individuals undergoing a severely energy‐restricted diet, the mean difference between the laboratory‐determined and monitor‐determined β‐hydroxybutyrate concentrations were only 0.01mM (*n* = 156 samples).^18^ Thirdly, the assessment of ketones in the urine with the use of a dipstick is subjective, as it involves comparison of the colour on the dipstick with the colour scale on the back of the packet. This limitation was contained as much as possible by limiting the assessment of urine acetoacetate levels to two trained researchers. Another limitation is that venous blood was collected from a cannula and not capillary blood collected via a finger prick. However, previous studies have shown that the concentration of β‐hydroxybutyrate tends to be higher in capillary blood than in venous blood.^26^ This suggests that the sensitivity of the urine dipsticks may be even lower than our observations, further supporting our conclusions that urine dipsticks are not accurate for detecting mild ketosis.

In summary, this study showed that urine dipsticks should not be recommended in clinical treatment protocols to monitor adherence to severely‐energy restricted diets, as they do not accurately reflect blood ketone levels typically found in people on these diets. If the detection of mild ketosis is indicated as a way to monitor or promote adherence, blood monitors should be used. Given the purported benefits of appetite suppression associated with mild ketosis, as well as the known benefits of self‐monitoring to improve weight loss success, it could be of benefit to investigate the use of blood monitors during a severely energy‐restricted diet to improve adherence.

## AUTHOR CONTRIBUTIONS

AAG conceived the idea for the substudy. AAG, AS and EIE contributed to the design of the sub‐study. AAG and CH implemented dietary intervention. RS oversaw the running of the trial and RS and SM collected the data. EIE conducted the statistical analysis. AAG and AS drafted the manuscript. All authors contributed to the interpretation of the findings, critical revision of the manuscript and approved the final version.

## CONFLICT OF INTEREST

AAG has received payment for oral presentations from the Pharmacy Guild of Australia and Nestlé Health Science. AS owns 50% of the shares in Zuman International Pty Ltd, which receives royalties for books she has written about adult weight management, and payments for presentations at industry conferences. She has also received presentation fees and travel reimbursements from Eli Lilly and Co, the Pharmacy Guild of Australia, Novo Nordisk, the Dietitians Association of Australia, Shoalhaven Family Medical Centres, the Pharmaceutical Society of Australia, and Metagenics, and she served on the Nestlé Health Science Optifast VLCD advisory board from 2016 to 2018. Prima Health Solutions, Sydney Australia, provided in‐kind support in the form of below‐cost KicStart™ VLED and a gift of associated adherence tools (shakers) for the TEMPO Diet Trial for which AS, AAG and RVS are investigators. Prima Health Solutions had no involvement in the design or analysis of the present research. This relationship with Prima Health Solutions was established after the most suitable product for the TEMPO Diet Trial had been determined.

## References

[1] Anderson JW , Konz EC , Frederich RC , Wood CL . Long‐term weight‐loss maintenance: a meta‐analysis of US studies. Am J Clin Nutr. 2001;74:579‐584. 11684524 10.1093/ajcn/74.5.579

[2] Parretti HM , Jebb SA , Johns DJ , Lewis AL , Christian‐Brown AM , Aveyard P . Clinical effectiveness of very‐low‐energy diets in the management of weight loss: a systematic review and meta‐analysis of randomized controlled trials. Obes Rev. 2016;17:225‐234. 26775902 10.1111/obr.12366

[3] Delbridge E , Proietto J . State of the science: VLED (Very Low Energy Diet) for obesity. Asia Pac J Clin Nutr. 2006;15:49‐54. 16928661

[4] Gibson AA , Franklin J , Pattinson AL , et al. Comparison of very low energy diet products available in Australia and how to tailor them to optimise protein content for younger and older adult men and women. Healthcare. 2016;4:71. 10.3390/healthcare4030071 PMC5041072 27657150

[5] Mustajoki P , Pekkarinen T . Very low energy diets in the treatment of obesity. Obes Rev. 2001;2:61‐72. 12119638 10.1046/j.1467-789x.2001.00026.x

[6] Christensen P , Meinert Larsen T , Westerterp‐Plantenga M , et al. Men and women respond differently to rapid weight loss: metabolic outcomes of a multi‐centre intervention study after a low‐energy diet in 2500 overweight, individuals with pre‐diabetes (PREVIEW). Diabetes Obes Metab. 2018;20:2840‐2851. 30088336 10.1111/dom.13466 PMC6282840

[7] Seimon RV , Wild‐Taylor AL , Keating SE , et al. Effect of weight loss via severe vs moderate energy restriction on lean mass and body composition among postmenopausal women with obesity: the TEMPO diet randomized clinical trial. JAMA Netw Open. 2019;2:e1913733‐e e1913733 31664441 10.1001/jamanetworkopen.2019.13733 PMC6824325

[8] Sumithran P , Prendergast LA , Delbridge E , et al. Ketosis and appetite‐mediating nutrients and hormones after weight loss. Eur J Clin Nutr. 2013;67:759‐764. 23632752 10.1038/ejcn.2013.90

[9] Chearskul S , Delbridge E , Shulkes A , Proietto J , Kriketos A . Effect of weight loss and ketosis on postprandial cholecystokinin and free fatty acid concentrations. Am J Clin Nutr. 2008;87:1238‐1246. 18469245 10.1093/ajcn/87.5.1238

[10] Gibson AA , Seimon RV , Lee CM , et al. Do ketogenic diets really suppress appetite? A systematic review and meta‐analysis. Obes Rev. 2015;16:64‐76. 25402637 10.1111/obr.12230

[11] Deemer SE , Plaisance EP , Martins C . Impact of ketosis on appetite regulation—a review. Nutr Res. 2020;77:1‐11. 32193016 10.1016/j.nutres.2020.02.010

[12] Laffel L . Ketone bodies: a review of physiology, pathophysiology and application of monitoring to diabetes. Diabetes Metab Res Rev. 1999;15:412‐426. 10634967 10.1002/(sici)1520-7560(199911/12)15:6<412::aid-dmrr72>3.0.co;2-8

[13] Gibson AA , Sainsbury A . Strategies to improve adherence to dietary weight loss interventions in research and real‐world settings. Behav Sci (Basel, Switzerland). 2017;7(3):44 10.3390/bs7030044 PMC5618052 28696389

[14] Dansinger ML , Gleason JA , Griffith JL , Selker HP , Schaefer EJ . Comparison of the Atkins, Ornish, Weight Watchers, and Zone diets for weight loss and heart disease risk reduction: a randomized trial. JAMA. 2005;293:43‐53. 15632335 10.1001/jama.293.1.43

[15] Alhassan S , Kim S , Bersamin A , King AC , Gardner CD . Dietary adherence and weight loss success among overweight women: results from the A TO Z weight loss study. Int J Obes (Lond). 2008;32:985‐991. 18268511 10.1038/ijo.2008.8 PMC4005268

[16] Corral PD , Bryan DR , Garvey WT , Gower BA , Hunter GR . Dietary adherence during weight loss predicts weight regain. Obesity (Silver Spring, Md). 2011;19:1177‐1181. 10.1038/oby.2010.298 PMC3215306 21164500

[17] Brewster S , Curtis L , Poole R . Urine versus blood ketones. Prac Diab. 2017;34:13‐15.

[18] Byrne HA , Tieszen KL , Hollis S , Dornan TL , New JP . Evaluation of an electrochemical sensor for measuring blood ketones. Diabetes Care. 2000;23:500‐503. 10857942 10.2337/diacare.23.4.500

[19] Arora S , Henderson SO , Long T , Menchine M . Diagnostic accuracy of point‐of‐care testing for diabetic ketoacidosis at emergency‐department triage: β‐Hydroxybutyrate versus the urine dipstick. Diabetes Care. 2011;34:852‐854. 21307381 10.2337/dc10-1844 PMC3064039

[20] Brooke J , Stiell M , Ojo O . Evaluation of the accuracy of capillary hydroxybutyrate measurement compared with other measurements in the diagnosis of diabetic ketoacidosis: a systematic review. Int J Environ Res Public Health. 2016;13 837 10.3390/ijerph13090837 PMC5036670 27563914

[21] MacLeod's D. Ketosis 2017 [Available from: http://www.drmacleods.com.au/products/ketosis-for-weight-loss.]

[22] Shake It Practitioner Weight Management Program. The Shake It Practitioner Weight Management Program—An Overview 2019 [Available from: https://www.shake-it.com.au/en/Articles/About/WhatIsShakeIt.]

[23] Gibson AA , Seimon RV , Franklin J , et al. Fast versus slow weight loss: development process and rationale behind the dietary interventions for the TEMPO Diet Trial. Obes Sci Pract. 2016;2:162‐173. 27840689 10.1002/osp4.48 PMC5089659

[24] Hsu MSH , Harper C , Gibson AA , et al. Recruitment strategies for a randomised controlled trial comparing fast versus slow weight loss in postmenopausal women with obesity—the TEMPO Diet Trial. Healthcare (Basel, Switzerland). 2018;6(3):76 10.3390/healthcare6030076 PMC6163885 29986398

[25] Seimon RV , Gibson AA , Harper C , et al. Rationale and protocol for a randomized controlled trial comparing fast versus slow weight loss in postmenopausal women with obesity—the TEMPO Diet Trial. Healthcare (Basel, Switzerland). 2018;6(3):85 10.3390/healthcare6030085 PMC6165329 30036996

[26] Norgren J , Sindi S , Sandebring‐Matton A , et al. Capillary blood tests may overestimate ketosis: triangulation between three different measures of beta‐hydroxybutyrate. Am J Physiol Endocrinol Metab. 2020;318:E184‐e188. 31821040 10.1152/ajpendo.00454.2019

